# Advances of Artificial Intelligence in Anti-Cancer Drug Design: A Review of the Past Decade

**DOI:** 10.3390/ph16020253

**Published:** 2023-02-07

**Authors:** Liuying Wang, Yongzhen Song, Hesong Wang, Xuan Zhang, Meng Wang, Jia He, Shuang Li, Liuchao Zhang, Kang Li, Lei Cao

**Affiliations:** Department of Biostatistics, School of Public Health, Harbin Medical University, Harbin 150081, China

**Keywords:** artificial intelligence, machine learning, neoplasms, drug design, databases

## Abstract

Anti-cancer drug design has been acknowledged as a complicated, expensive, time-consuming, and challenging task. How to reduce the research costs and speed up the development process of anti-cancer drug designs has become a challenging and urgent question for the pharmaceutical industry. Computer-aided drug design methods have played a major role in the development of cancer treatments for over three decades. Recently, artificial intelligence has emerged as a powerful and promising technology for faster, cheaper, and more effective anti-cancer drug designs. This study is a narrative review that reviews a wide range of applications of artificial intelligence-based methods in anti-cancer drug design. We further clarify the fundamental principles of these methods, along with their advantages and disadvantages. Furthermore, we collate a large number of databases, including the omics database, the epigenomics database, the chemical compound database, and drug databases. Other researchers can consider them and adapt them to their own requirements.

## 1. Introduction

In recent years, many companies have ramped up their R&D (research and development) efforts for anti-cancer drugs [1]. There is a growing number of large and long-term clinical trials providing a possible therapeutic opportunity for more cancer patients [2,3]. Recently, the American Cancer Society announced that the three-year survival rate for lung cancer from 2014 to 2021 was raised from 21% to almost 31% [4]. The efficacy of targeted therapies and immunotherapeutics has been investigated in a variety of solid tumors [5]. Thus, a greater investment in targeted therapies and immunotherapeutics to realize the benefits of precision medicine will benefit the long-term survival of cancer patients [6,7,8].

The anti-cancer drug design and discovery workflow comprises target recognition, hit exploration, hit-to-lead development, lead optimization, preclinical drug candidate identification, and preclinical and clinical research [9,10,11]. Despite the improvements in tumor biotechnology and the advances in cancer mechanism research, the development of novel and effective anti-cancer drugs from scratch remains an arduous, expensive, and time-consuming process [12] that will require close multidisciplinary collaborations, including medicinal chemistry, computational chemistry, biology, pharmacology, and clinical research [13]. Statistically, it can take more than 10–17 years and almost 2.8 billion dollars to bring a new drug into clinical practice [14,15]. Apart from that, only 10% of the tested compounds in clinical trials reach the market [16].

It is especially difficult to design anti-cancer drugs due to challenges such as undruggable targets [17], chemoresistance in oncology [18], tumor heterogeneity [19], and metastasis [20]. The conventional drug design approaches may seem poorly effective. With so many challenges still to be faced, the treatment effects among cancer patients are actually suboptimal. Thus, more effective anti-cancer drug design strategies are urgently needed. They will reduce the cost of drug development and the time required for clinical trials. They can also help increase the global life expectancy and improve human health [4].

Computer-aided drug design (CADD) is a method that began in the early 1980s [21]. The use of computer-aided methods to guide drug screening is emerging as an important component in the practice of drug design [22,23,24,25]. This approach enabled medicinal chemists to calculate the interactions between a ligand and receptors and to design and optimize lead compounds by computer simulation [26]. The typical role of CADD in drug design is to screen out large compound libraries into smaller clusters of predicted active compounds based on computational chemistry. It can greatly speed up the process of anti-drug design and save a huge amount of time and money [27].

**Figure 1:**
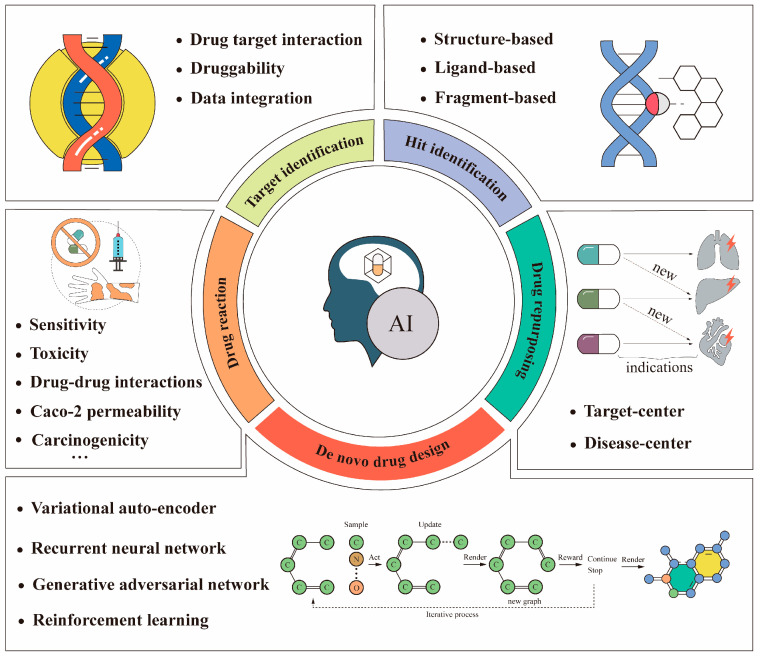
Some applications of artificial intelligence in anti-cancer drug design. The bottom (de novo drug design) is usually implemented using the deep learning-based models listed above. Recently, reinforcement learning has been used often. The above workflow example of a graphical chemical structure with an O–C–O connection is an iterative chemical graph generation process [33].

In the context of the rapid development of computer hardware and artificial intelligence techniques, researchers in academia and the pharmaceutical industry are turning to artificial intelligence to improve drug design processes [28]. Artificial intelligence (AI) refers to the simulation of human intelligence in machines that are programmed to think and act like humans [15]. A common presumption about artificial intelligence is that its goal is to build machines with a similar capacity for “understanding” [29]. Artificial intelligence is now used in many applications for cancer research, such as image classification of abnormal cancer cells [30], prediction of target protein structures [31], and prediction of drug–protein interactions [32]. These studies demonstrate that artificial intelligence techniques have the power to revolutionize anti-cancer drug design processes. Some applications using artificial intelligence in anti-cancer drug design processes are illustrated in Figure 1. This paper reviewed some of the advances in anti-cancer drug design based on artificial intelligence, presented some of the most classic examples, and clarified the fundamental principles of these methods.

## 2. Method

The present study is a narrative review of the literature. We performed searches in the US National Library of Medicine (PubMed) to find original articles. The search strategy used in PubMed is shown in Table 1. We mainly focused on the articles and reviews published in the past decade. The last search of the present narrative review was performed on 10 December 2022.

## 3. Artificial Intelligence in Anti-Cancer Drug Target Identification

The identification of drug–target interactions (DTIs) is the initial step in anti-cancer drug design. The strength of drug–target binding is often described by binding affinity constants, including indicators such as a dissociation constant (Kd), an inhibition constant (Ki), and a half-maximal inhibitory concentration (IC50) [34]. Since the experimental determination of DTIs is a time-consuming and expensive process, its computational prediction is of great interest. Accurate and effective DTI predictions can greatly aid drug development and accelerate lead or hit compound discovery.

### 3.1. Artificial Intelligence Efficiently Elevates the Prediction Accuracy of DTI

Traditionally, the computational methods for DTI predictions have included molecular docking simulation and machine learning-based methods. However, these studies would be expensive, time-consuming, and difficult to conduct without knowing the 3D structures of the drug targets. Peng et al. developed a novel end-to-end learning framework based on heterogeneous graph convolutional networks (EEG)-DTI for DTI predictions. A graph convolutional network-based model was used to learn the low-dimensional feature representations of drugs and targets and predict the DTI based on the learned features. It achieved a promising DTI prediction performance even when the 3D structures of the drug targets were not used [35]. To further improve the prediction performance, Shao et al. considered the DTI prediction as a link prediction problem and proposed an end-to-end model based on the heterogeneous graph with attention mechanism (DTI-HETA), which outperformed the state-of-the-art models [36]. Meanwhile, to address the explanation problem of deep learning, Yang et al. proposed a drug–target interaction prediction method based on mutual learning mechanisms without 3D structural information and with explanation [37].

### 3.2. Artificial Intelligence Could Integrate Data from Multiple Sources to Help with Anti-Drug Target Identification

Drug target identification is a key step in drug development. However, most previous studies were confined to a single data type and did not integrate multiple data types. Thus, they were vulnerable to data-specific noise and needed to be improved in terms of practicality and accuracy [38]. Recently, there has been a growing number of methods within similarity-based or data-driven frameworks that attempt to use artificial intelligence to improve the predictive power by integrating multiple different data types. Madhukar et al. developed a Bayesian-based machine learning method (BANDIT), which achieved approximately 90% target prediction accuracy on more than 2000 small molecules by integrating six types of data, including growth inhibition data, gene expression data, adverse reaction data, chemical structure data, and drug data [39]. Olayan et al. proposed a method named DDR to investigate how to predict drug–target interactions more efficiently by using data from different sources, which included eight drug similarity networks and eight target similarity networks. The drug similarity networks included the following: gene expression similarity, disease-based similarity, drug side effect-based similarity, chemical structure fingerprint-based similarity, etc. The target similarity networks included the following: gene ontology-based similarity, protein sequence-based similarity, etc. [40]. The above studies illustrated that integrating data from multiple sources through artificial intelligence could increase the biological explanation of drug target prediction and prediction accuracy.

### 3.3. Artificial Intelligence Could Help Predict the Druggability of Anti-Cancer Drug Targets

The selection of drug targets is also a very critical step in the cancer drug design process, and it has a great impact on the success rate of later clinical trials. Therefore, many related methods were developed. Raies et al. proposed a prediction model called DrugnomeAI to address the problem of targeted drug synthesis. The stochastic semi-supervised machine learning framework was used to develop DrugnomeAI for predicting the druggability of drug targets in the human exome. It also demonstrated how the application of DrugnomeAI can predict the druggability of drug targets in oncology diseases [41]. In recent years, an increasing number of studies have identified synthetic lethality (SL) as a promising approach for the discovery of anticancer drug targets [42]. However, the wet experimental screening for SL has problems, including high costs, batch effects, and off-target results. Wang et al. designed a new model based on a graph neural network (GNN) called KG4SL. It incorporates knowledge graph (KG) messaging into a graph neural network prediction. The experimental results demonstrated a significant beneficial effect of incorporating KG into the GNN for SL predictions [43]. The Table 2 below lists some of the methods for anti-cancer drug target identification based on artificial intelligence that have been developed in recent years.

## 4. Artificial Intelligence in the Screening of Anti-Cancer Drug Hit Compounds

After the identification of therapeutic targets for anti-cancer drugs, we need to screen for anti-cancer drug hit compounds, which are molecules with initial activities against a specific target or linkage of action [49]. The discovery of computer-aided hit compounds is mainly through high-throughput screening. High-throughput screening can be performed in the following two ways: structure-based screening and ligand-based screening [50]. Fragment-based screening methods are also effective for the discovery of hit compounds, as shown in recent studies [51]. High-throughput screening techniques have been highly successful in many R&D projects, but the efficiency of screening compounds by the millions has reached a bottleneck, and the cost is also significant [52]. With the proliferation of GPUs, increased computer power, and the rapid development of artificial intelligence technologies, more virtual hit compound screening tools have been developed to enrich the drug design toolkit.

### 4.1. Structure-Based Screening of Hit Compounds Using Artificial Intelligence

Structure-based virtual screening uses docking and scoring to select molecules that have good binding affinity with a target protein [53]. This strategy is an important tool for anti-cancer drug design, but many of the current docking procedures are time-consuming and pose challenges for large-scale virtual screening. Lu et al. accelerated the evaluation process through structure screening with the help of deep learning models. They constructed a deep learning model to predict molecular docking scoring [54]. Yasuo et al. used artificial intelligence to propose a new structure-based virtual screening method for hit compounds, called SIEVE-Score, which provided substantial improvements over other state-of-the-art virtual screening methods [55].

### 4.2. Ligand-Based Screening of Hit Compounds Using Artificial Intelligence

Ligand-based screening is based on taking small molecules with known activities and searching for structures with similar physical or chemical characteristics in a compound library as candidates. Krasoulis et al. proposed an end-to-end method called DENVIS, a scalable and novel algorithm for high-throughput screening using graphical neural networks with atomic and surface protein pocket features. By conducting experiments on two benchmark databases, DENVIS was much faster than other models [56]. This method was not only advantageous in terms of speed and had an impressive success rate, but it was also easy to use. Generally, most of these methods could only receive one representative molecular structure as a search template [57], which may result in data waste. To address this problem, Hutter developed a cumulative molecular fingerprinting algorithm that can take all structure data into account in the calculation, effectively improving the utilization of experimental data and achieving an organic combination of molecular fingerprinting and experimental data. It inherited the speed advantage of the former method with higher information utilization [58].

### 4.3. Fragment-Based Screening of Hit Compounds Using Artificial Intelligence

In recent years, the rise of emerging technologies such as high-throughput screening (HTS) and combinatorial chemistry (CC) has led to the gradual systematization of drug discovery from the randomized screening of known drugs [59]. These methods can significantly increase the speed of drug discovery and shorten the process of new drug development, but the high cost of screening has also increased the research burden on small drug development companies and research institutions. Therefore, many researchers are focusing on fragment-based drug design (FBDD) [60]. Compared with the traditional screening methods, FBDD starts with small molecular fragments, which greatly reduces the size of the required screening compound library, circumvents the undesirable ADMET properties of molecules, and enhances the diversity of the designed structures [61]. In addition, FBDD has potential advantages for the drug design of difficult targets and has gradually developed into a mainstream drug design method in small drug development companies and research units [62]. To ligate fragments rationally, it is necessary to know where the fragments bind in a pocket. Currently, the main computational prediction methods are molecular docking, functional group mapping, and molecular structure splitting and reconstruction. These methods are more or less limited by computational costs and manual judgement and cannot fully utilize the structural data of protein–ligand complexes. To solve this problem, Didier Rognan’s group proposed the method POEM, which is based on the recognition and matching of the pocket environment in which the fragments are located [63]. Another challenge of FBDD is linking fragments to generate interest libraries of compounds for specific drug targets. To address this issue, Yang et al. proposed a model based on automatic fragment linking with deep conditional transformer neural networks called SyntaLinker [64]. Caburet et al. screened the activity of NDM-1 β-lactamase inhibitors using the FBDD method. They finally found 37 fragments for pharmacophore establishment, which was proven to be accurate and efficient. Table 3 lists all of these methods [65].

## 5. Artificial Intelligence in De Novo Anti-Cancer Drug Design

The chemical space of drug-like molecules is extremely vast; the number is estimated to be 10^23^~10^60^ [66]. Therefore, it is nearly impossible to completely mine the entire chemical space using computational methods. In this context, finding specific lead compounds in the vast chemical space is a major challenge. With the rapid development of computational power and experimental techniques, high-throughput screening (HTS) and virtual screening (VS) methods can effectively evaluate molecules in large compound libraries with a wide variety of filters [67,68].

However, both traditional HTS and vs. methods that are based on molecular docking can only screen the known compound library to find molecules that satisfy specific properties [69]. De novo drug design and virtual screening are very similar in the sense that they both search for molecules that meet specific requirements in the chemical space. However, their processes are very different. Instead, de novo drug design is a molecule generation method that generates and optimizes a molecule by ultimately using artificial intelligence [70]. Molecular generation methods include variational auto-encoders (VAEs), the recurrent neural network (RNN), the generative adversarial network (GAN), and deep reinforcement learning (DRL) [71].

### 5.1. Application of Variational Auto-Encoder to De Novo Design of Anticancer Drugs

The variational auto-encoder (VAE) is an important type of generative model that was proposed by Diederik P. Kingma and Max Welling in 2013 [72]. Born et al. constructed a hybrid VAE model to generate candidate molecules with anti-cancer drug properties. The model was able to generate molecules with strong inhibitory effects against specific diseases. The generated molecules were similar to existing drugs in terms of structure, synthesizability, and solubility [73]. Hong et al. proposed a molecular structure tree generation model in which the molecules were generated by gradually adding substructures [74]. The proposed model was based on a VAE architecture, which used an encoder to map molecules into the latent vector space and then built an autoregressive generative model as a decoder to generate new molecules from a Gaussian distribution. It showed that the model can generate efficient and new molecules and that the optimized model can effectively improve the properties of the molecules. Samanta et al. proposed the NEVAE method, which solved the problems of current methods. For instance, existing models can only generate molecules with the same number of atoms but fail to utilize a large number of macromolecules in the training process, limiting the diversity of the generated molecules. In addition, they cannot provide the spatial coordinates of the generated atoms [75].

### 5.2. Application of the Recurrent Neural Network to De Novo Design of Anti-Cancer Drugs

The recurrent neural network (RNN) model uses basic units, such as atoms or fragments of molecules, as the basic vocabulary and generates molecules in a temporal order. The output probability of the next atom character generated by the RNN model depends on the previous generated atom. The RNN-based model has been widely used to process time-series-related data, such as language, text, video, etc. [76]. Grisoni et al. proposed a new bidirectional RNN molecular generation model, or BIMODAL, that can be used for SMILES generation and data enhancement [77]. The model performed bidirectional molecular design by alternate learning, and the model was compared with other bidirectional RNNs. BIMODAL was promising in terms of molecular novelty, backbone diversity, and chemical and biological relevance of the generated molecules and was superior to the state-of-the-art methods [78,79]. To address problems such as the poor performance of DL on small training datasets, Krishnan et al. designed a de novo drug design method based on RNN generative models and migration learning to generate molecules with not only the desired drug-like properties but also target specificity [80]. In addition, Moret et al. combined the RNN generation model with three optimization methods, namely data augmentation, temperature sampling, and transfer learning. This method can generate new molecules with the desired properties with a small amount of data [81].

### 5.3. Application of Generative Adversarial Network to De Novo Design of Anti-Cancer Drugs

The generative adversarial network (GAN) is an unsupervised learning method proposed by Goodfellow in 2014. It consists of the following two networks: the generative network G, which is used to fit the data distribution, and the discriminative network D, which is used to determine whether the input is “real” or not. In the training process, the generative network G tries to “cheat” D by accepting random noise to imitate the real images in the training set, while D tries to distinguish the real data from the output of the generative network as much as possible, thus forming a game process between the two networks. Ideally, the game results in a generative model that can be “faked” [82]. Maziarka et al. proposed the Mol-Cycle GAN method. Mol-Cycle GAN is a conditional generative adversarial network-based method for de novo drug design and synthesis optimization of molecules through a generative model. It can solve the problem of difficult-to-synthesize compounds given a starting molecule. It can also generate molecules with similar structures and desired properties [83]. ABbbasi et al. proposed a feedback-based GAN framework that implemented an optimization strategy by connecting an encoder–decoder, a GAN, and a predictor depth model with a feedback loop. The results showed that molecules with high binding affinity can be generated by the GAN optimization model [84].

### 5.4. Application of Deep Reinforcement Learning to De Novo Design of Anti-Cancer Drugs

Even though a variety of drug generation models have been developed, they all focus on the following two points: molecular representation and optimization strategies [71]. Deep reinforcement learning (DRL) is an artificial intelligence technique that combines the perceptual capabilities of deep learning with the decision-making capabilities of reinforcement learning to solve decision-making problems in high dimensional and state spaces [85]. A novel computational strategy, called ReLeaSE, was proposed by Tropsha for designing molecules with desired properties from scratch. ReLeaSE was built on deep learning (DL) and reinforcement learning (RL) methods by integrating two deep neural networks (generative and predictive), which were trained to generate novel libraries of molecules with specified properties [86]. Goel et al. combined RNN and reinforcement learning to propose a molecule generation model named MoleGuLAR that can perform multi-objective optimization of molecules in terms of drug-like properties and binding affinity. In particular, they proposed a new alternating reward strategy where the reward function changes dynamically as different molecules are generated, allowing the model to alternately explore different chemical intervals and sample more reasonable molecules [87]. Table 4 shows some of these methods.

## 6. Artificial Intelligence in Anti-Cancer Drug Repurposing

Effective identification of new indications from approved or established clinical drugs plays a critical role in drug discovery. Such a process is also known as drug repositioning. Despite tremendous efforts in academic and pharmacological research worldwide, current anti-cancer therapies have achieved success in only a few tumor types. The application of drug repositioning in tumor therapies is a hot topic in current research. In theory, repurposing is faster, safer, easier, and less expensive than the known barriers to developing new molecular entities. Opportunities for drug repurposing are often based on incidental observations or time-consuming preclinical drug screens that are not usually hypothesis-driven. Indeed, the widespread use of histology technologies, improved electronic medical record systems, improved data storage, data meaning, machine learning algorithms, and computational modeling have provided unprecedented knowledge of the biological mechanisms of cancer and drug modes of action, providing broad availability of both disease-related and drug-related data. Drug repositioning strategies are often categorized as “target-center” and “disease-center” methods for predicting unknown drug–target and drug–disease interactions.

### 6.1. Artificial Intelligence in Anti-Drug Repositioning Based on the Interaction between a Drug and a Target

Many artificial intelligence-based methods have been used to predict drug–target relationships, as described above. At present, predicting drug–target relationships is one of the main approaches for drug repurposing. To achieve personalized drug repurposing using genomic information, Cheng et al. developed a genome-wide localization system network algorithm (GPSnet) [88]. This method uses patient-specific DNA and RNA sequencing profiles of specific targets to obtain disease modules for repurposing drugs. They validated that the approved arrhythmia and heart failure drug Ouabain specifically targets the HIF1α/LEO1-mediated cellular metabolic pathways in lung adenocarcinomas, showing potential anti-tumor activities. Wang et al. proposed a deep learning framework through kernel-based data integration, known as DeepDRK [89]. The model was trained on over 20 000 pairs of pan-cancer cell line anti-cancer drug pairs. These pairs were characterized by using kernel-based similarity matrices that integrate multi-source and multi-omics data, including genomics, transcriptomics, epigenomics, chemical properties of compounds, and known drug–target interactions. They provided a computational approach to predict cancer cell responses to drugs by integrating pharmacogenomic data, offering an alternative approach to repurposing drugs in cancer precision therapy.

### 6.2. Artificial Intelligence in Anti-Drug Repositioning Based on the Interaction between Drugs and Diseases

Predicting drug–disease interactions is essential for disease-centric drug repurposing. The current identification of drug–disease interactions is mainly based on similarity and network, respectively. For the similarity-based methods, Zhang et al. proposed a multiscale drug–disease topology learning framework (MTRD). By learning the representative properties of drug–disease, this method explored a new therapeutic effect of existing drugs based on the relevant similarity and association information of drug–disease node pairs. [90]. Jarada et al. proposed a novel framework based on deep learning, known as SNF-NN, to predict new drug–disease interactions using drug-related similarity information, disease-related similarity information, and known drug–disease interactions [91]. Luo et al. proposed a new computational method named MBiRW [92], which uses combined similarity measurements and a birandom walk (BiRW) algorithm to identify potential new indications for known drugs. This method was based on the assumption that similar drugs are usually associated with similar diseases. Moreover, Sadeghi et al. proposed a new model named DR-HGNN for drug repositioning using multiple labeling of heterogeneous graph neural networks [93]. Doshi et al. proposed a graph neural network-based drug repositioning model called GDRnet [94], which was able to efficiently screen the database for existing drugs and predict their unknown therapeutic effects. Table 5 shows some of the methods mentioned above.

## 7. Artificial Intelligence-Assisted Accurate Prediction of Anti-Cancer Drug Reactions

Drug reactions are related to their ADMET properties, which may influence drug sensitivity, drug toxicity, and drug–drug interactions [95,96]. The accurate prediction of drug reactions can effectively increase the success rate of clinical trials and improve patient outcomes. With the rapid development of artificial intelligence technologies, more and more related studies are being proposed at the drug design stage using artificial intelligence techniques.

### 7.1. Artificial Intelligence Aids in Predicting the ADMET Properties of Anti-Cancer Drugs

To explore drug reactions, the ADMET properties should be accurately predicted first. Several ADMET properties, including Caco-2 permeability, carcinogenicity, blood–brain barrier permeability, and plasma protein binding, are included in previous studies. For instance, Selvaraj et al. reviewed the applications of various machine learning models, such as SVM regression and partial least squares (PLSs), for the prediction of the Caco-2 permeability coefficient [97]. Li et al. proposed a DeepCarc model to predict the carcinogenicity of small molecules using deep learning-based model-level representations [98]. Vatansever et al. reviewed the current state-of-the-art methods in AI-guided central nervous system (CNS) drug discovery, focusing on the blood–brain barrier permeability prediction [99]. To predict the plasma protein binding of a drug, Mulpuru et al. built a prediction model of a fraction of unbound drug in human plasma using a chemical fingerprint and a freely available AutoML framework [100].

### 7.2. Artificial Intelligence Aids in Predicting Anti-Cancer Drug Sensitivity

Anti-cancer drug sensitivity predictions are important in guiding the enrollment of those patients who may benefit from specific treatments. Chawla et al. developed a deep neural network named Precily, which uses gene expression data to predict drug sensitivity for cancer therapy. The model combines the structural properties of drugs with the pathway specificity of gene expression as features to train the model [101]. Eliseo Papa et al. built a recommendation system based on the BIKG knowledge graph to predict drug sensitivity and identified effective patient subgroups early in clinical trials [102]. Gerdes et al. proposed a model called DRUML, which uses omics data to rank over 400 drugs based on their anti-tumor cell proliferation efficacy. The results showed that DRUML can accurately rank anti-cancer drugs based on their efficacy [103].

### 7.3. Artificial Intelligence Aids in Predicting Toxicity of Anti-Cancer Drugs

Drug toxicity is a central issue to be considered in the drug development process. Recently, Wang et al. proposed a machine learning classifier that combines chemical structure (CS) and gene expression (GE) features. In addition, they prioritized the adverse effects of approved drugs and preclinical small-molecule compounds. The results showed that integrating GE data with drug CSs can significantly improve the predictability of adverse effects [104]. However, most of the current studies only predict the occurrence of adverse drug reactions, not their intensity or frequency. To address this issue, Zhao et al. designed a novel graphical attention model for predicting drug side effect frequency from multi-view data. The computational results showed the best performance on the benchmark dataset, illustrating effectiveness in predicting the frequency of drug side effects [105].

### 7.4. Artificial Intelligence Can Predict Drug–Drug Interactions

Zhu et al. proposed a unified multi-attribute discriminative representation learning (MADRL) model for DDI predictions. MADRL uses a generative adversarial network (GAN) to capture intra-attribute specificity information of DDI attributes and uses them for DDI predictions. The effectiveness of the MADRL algorithm was validated on a publicly available dataset [106]. Most methods for predicting drug–drug interactions only predict whether there is an interaction between two drugs, but it is more relevant to investigate the hidden mechanisms behind DDIs. Therefore, Zhang et al. proposed a deep learning method (DDIMDL) that used multiple drug features to predict the types of drug–drug interaction events and explored their hidden mechanisms [107]. To further increase the model’s accuracy and biological explanation, Chen et al. developed 3DGT-DDI, which consists of a 3D graph neural network and a pre-trained textual attention module. The innovation of the method is that it utilizes a 3D molecular graph structure and location information to enhance the prediction ability of DDIs. The experiments showed that the prediction performance of 3DGT-DDI outperformed other baseline models [108]. Table 6 table shows some of the methods mentioned above.

## 8. Data Sources of Artificial Intelligence to Anti-Cancer Drug Designs

A large number of artificial intelligence-based algorithms, including deep learning, have become powerful tools in AI-assisted anti-cancer drug design [110,111]. Scientists are developing algorithms that can learn and analyze large amounts of data with superhuman efficiency to speed up the anti-cancer drug design process [112]. However, artificial intelligence is not universal and requires large amounts of reliable data or training experiences [113]. Nowadays, there are some specific databases for artificial intelligence-based anti-cancer drug design. They are listed in Table 7.

## 9. Successful Cases Applying AI in Anti-Cancer Drug Design

To depict how AI facilitates the development of anticancer drugs, we list some of the anticancer drugs that have successfully entered human phase 2/3 clinical trials in the last 5 years in Table 8. For instance, Recursion identified REC-2282 as a potential candidate for the treatment of diseases caused by mutations in the NF2 gene through its proprietary AI-driven drug discovery platform, Recursion OS. REC-2282 is a permeable, orally bioavailable, small-molecule HDAC inhibitor that is being developed for the treatment of meningiomas with mutations in the NF2 gene. This molecule appears to be well tolerated, including in patients that have been administering it over several years, and different from other HDAC inhibitors in that it may reduce cardiotoxicity. It was granted both orphan drug status and fast-track status by the U.S. FDA [114]. Relay Therapeutics developed the FGFR2-specific inhibitor RLY-4008 by analyzing the dynamic balance of protein conformations through an artificial intelligence platform. Preclinical studies have shown that RLY-4008 exhibits high selectivity for FGFR2 targets in cancer cell lines, shrinking tumors with minimal impact on other targets [115]. Breg developed a new drug, BPM 31510, through an artificial intelligence platform that is currently in clinical testing. The drug restructures the metabolism of cancer cells so that patients do not have to undergo chemotherapy, allowing cancer cells to die naturally [116]. EXS-21546 is an AI-designed A2A receptor antagonist. Some tumors produce high levels of adenosine, which binds to and activates the A2A receptors on immune cells, thereby inhibiting the anti-tumor activity of the immune system [117]. PHI-101 is an orally available, selective checkpoint kinase 2 (Chk2) inhibitor designed by an AI-driven drug discovery platform [118].

## 10. Conclusions and Prospects of Future Challenges

This review focuses on work that has been performed in the past decade on anti-cancer drug design based on artificial intelligence. Compared to other reviews, our study collated a large number of databases and source codes. It will offer some guidelines for other researchers to apply to their own research. This means our review has great practicality.

Artificial intelligence (AI) has strong logical reasoning and independent learning abilities that can simulate the thinking process of a human brain. AI technologies, such as machine learning, can profoundly optimize the existing anti-cancer drug research paradigm. In recent years, AI has already made unique contributions to the development and treatment of anti-cancer drugs. Artificial intelligence can accelerate the discovery of new drug molecules and the synthesis of more desirable drug molecules. This process may greatly accelerate the development of anti-cancer drugs. It is believed that artificial intelligence will be a powerful driving force for human cancer research and treatment in the future. However, AI also has several limitations, including a high dependence on data and a limited explanation. The “black box” behind traditional AI models prevents scientists from using algorithms for hypothesis validation and mining the logic behind the data. Moreover, in the drug development process, predicting the underlying logic behind a model is critical to designing the right drug molecules. In the future, interpretable AI models will be the new development direction, and the close combination of data and computation will be a feature of AI-assisted cancer drug development. We believe that AI will bring profound changes to anti-cancer drug designs.

Our study is also subject to certain limitations. For instance, we only focused on articles published in the last ten years. In addition, the search was limited to the database of PubMed. We will address these limitations in future studies.

## Figures and Tables

**Table 1:** Search strategies used in the US National Library of Medicine (PubMed), according to selected descriptors.

Strategy	Descriptors Used
#1	(“cancer” [Title/Abstract] AND “artificial intelligence” [Title/Abstract] AND “drug” [Title/Abstract]) AND (y_10[Filter])
#2	(“cancer” [Title/Abstract] AND “drug discovery” [Title/Abstract] AND “AI” [Title/Abstract]) AND (y_10[Filter])
#3	(“cancer” [Title/Abstract] AND “drug design” [Title/Abstract] AND “machine learning” [Title/Abstract]) AND (y_10[Filter])
#4	(“database” [Title/Abstract] AND “drug” [Title/Abstract] AND “artificial intelligence” [Title/Abstract]) AND (y_10[Filter])

**Table 2:** Methods for anti-cancer drug target identification based on artificial intelligence.

Model	Data Source	Code	References
EEG-DTI	Luo dataset [44], Yamanishi dataset [45]	https://github.com/MedicineBiology-AI/EEG-DTI (5 July 2022)	[35]
DTI-HETA	Yamanishi dataset	https://github.com/ZhangyuXM/DTI-HETA (13 October 2022)	[36]
ML-DTI	Metz dataset, KIBA dataset, Davis dataset, [46,47,48], Drugbank	https://github.com/guaguabujianle/ML-DTI.git (19 June 2021)	[37]
DDR	Yamanishi dataset, KEGG BRITE, BRENDA, SuperTarget, DrugBank	https://bitbucket.org/RSO24/ddr/(22 November 2017)	[40]
DrugnomeAI	TCRD, StringDB, CTDbase, InterPro, OMIM	https://github.com/astrazeneca-cgr-publications/DrugnomeAI-release (4 November 2022)	[41]
KG4SL	SynLethDB	https://github.com/JieZheng-ShanghaiTech/KG4SL (12 September 2021)	[43]

**Table 3:** Artificial intelligence-based screening methods of anti-cancer drug hit compounds.

Model	Data Source	Code	References
SECSE	PDB	http://github.com/KeenThera/SECSE (15 July 2022)	[54]
SIEVE-Score	ChEMBL, ZINC	https://github.com/sekijima-lab/SIEVE-Score (15 November 2019)	[55]
DENVIS	PDB, DUD-E, LIT-PCBA	https://github.com/deeplab-ai/denvis (3 October 2022)	[56]
DMMFP	ChEMBL, DUD-E, ZINC	https://github.com/michahutter/multimolecule_fingerprints (28 April 2022)	[58]
POEM	ChEMBL, ZINC, PDB	https://github.com/kimeguida/POEM (30 December 2022)	[63]
SyntaLinker	ChEMBL	https://github.com/YuYaoYang2333/SyntaLinker (18 June 2021)	[64]

**Table 4:** Methods for de novo anti-cancer drug design through artificial intelligence.

Model	Data Source	Code	References
PaccMannRL	TCGA, ChEMBL, GDSC, CCLE	https://github.com/PaccMann/ (10 February 2022)	[73]
ACGT	QM9, ZINC	https://github.com/gicsaw/ARAE_SMILES (14 October 2022)	[74]
NEVAE	QM9, ZINC	https://github.com/Networks-Learning/nevae (22 November 2019)	[75]
BIMODAL	ChEMBL	https://github.com/ETHmodlab/BIMODAL (3 June 2020)	[77]
Mol-CycleGAN	ChEMBL, ZINC	https://github.com/ardigen/mol-cycle-gan (6 February 2019)	[83]
GAN-Drug-Generator	ChEMBL, ZINC	https://github.com/larngroup/GAN-Drug-Generator (13 April 2022)	[84]
ReLeaSE	ChEMBL, ZINC	https://github.com/isayev/ReLeaSE (9 December 2021)	[86]
MoleGuLAR	ChEMBL, ZINC	https://github.com/devalab/MoleGuLAR (21 October 2021)	[87]

**Table 5:** Methods for anti-cancer drug repurposing based on artificial intelligence.

Model	Data Source	Code	References
GPSnet	DrugBank, TTD, PharmGKB, ChEMBL, BindingDB, UniProt, TCGA	https://github.com/ChengF-Lab/GPSnet (16 December 2018)	[88]
DeepDRK	CTRP, GDSC, TCGA, DrugBank, KEGG	https://github.com/wangyc82/DeepDRK (16 January 2021)	[89]
MBiRW	Drugbank, OMIM	http://github.com//bioinfomaticsCSU/MBiRW (19 December 2016)	[92]
DR-HGNN	Drugbank, CTD, SIDER	https://github.com/sshaghayeghs/DR_HGNN (26 April 2022)	[93]
GDRnet	Drugbank, Hetionet, GNBR, STRING, IntAct, DGIdb	https://github.com/siddhant-doshi/GDRnet (27 December 2021)	[94]

**Table 6:** Methods for prediction of cancer drug reactions based on artificial intelligence.

Model	Data Source	Code	References
DeepCarc	CPDB, Pubchem, Drugbank	https://github.com/TingLi2016/DeepCarc (6 July 2022)	[98]
Precily	CCLE, MSigDB, GDSC, Pubchem	https://github.com/SmritiChawla/Precily (26 August 2022)	[101]
DRUML	PharmacoDB, DepMap portal, PRIDE dataset, DrugBank, ChEMBL	https://github.com/CutillasLab/DRUMLR (24 March 2022)	[103]
MGPred	SIDER, STITCH, DrugBank, PubChem	https://github.com/zhc940702/MGPred (6 May 2018)	[105]
DLADE	cTAKES, EHR and PubMed article	https://github.com/qinxiao (7 October 2022)	[109]
MADRL	KEEG, SIDER, CTD, DrugBank	https://github.com/AdverseDDI/MADRL (18 January 2022)	[106]
DDIMDL	DrugBank, KEGG	https://github.com/YifanDengWHU/DDIMDL (1 May 2021)	[107]
3DGT-DDI	DrugBank, DDI extraction 2013	https://github.com/hehh77/3DGT-DDI (21 February 2022)	[108]

**Table 7:** Different data sources for anti-cancer drug design.

Database	Website
BindingDB	https://www.bindingdb.org/bind (24 December 2022)
BRENDA	https://www.brenda-enzymes.org/ (1 February 2023)
CCLE	https://sites.broadinstitute.org/ccle/ (23 December 2019)
chEMBL	https://www.ebi.ac.uk/chembldb (12 July 2022)
CPDB	https://www.nlm.nih.gov/databases/download/cpdb.html (12 October 2022)
CPTAC	https://proteomics.cancer.gov/programs/cptac (7 February 2023)
CTDbase	http://ctdbase.org (1 February 2023)
CTRP	https://portals.broadinstitute.org/ctrp.v2.1/ (7 February 2023)
DepMap	https://depmap.org/portal/ (14 December 2022)
DGIdb	www.dgidb.org. (21 October 2020)
Drugbank	https://www.drugbank.com/ (7 February 2023)
DUD-E	http://dude.docking.org/ (14 July 2012)
GDSC	https://www.cancerrxgene.org/ (July 2022)
GEO	https://www.ncbi.nlm.nih.gov/geo/ (7 February 2023)
HCA	https://data.humancellatlas.org/ (7 February 2023)
Hetionet	https://het.io/ (7 February 2023)
IntAct	https://www.ebi.ac.uk/intact/ (December 2021)
InterPro	https://www.ebi.ac.uk/interpro (November 2022)
JingleBells	http://jinglebells.bgu.ac.il/ (7 February 2023)
KEGG	https://www.genome.jp/kegg/ (1 January 2023)
LIT-PCBA	https://drugdesign.unistra.fr/LIT-PCBA/ (7 February 2023)
MSigDB	https://www.gsea-msigdb.org/gsea/msigdb/ (August 2022)
OMIM	https://www.omim.org. (5 February 2023)
Open Targets	https://www.opentargets.org/ (19 January 2023)
PDB	https://www.rcsb.org/docs/general-help/organization-of-3d-structures-in-the-protein-data-bank (31 August 2022)
PharmacoDB	https://pharmacodb.ca/ (7 February 2023)
PharmGKB	https://www.pharmgkb.org/ (7 February 2023)
portal	https://help.hcltechsw.com/digital-experience/9.5/plan/db_domains.html (7 February 2023)
PubChem	https://pubchem.ncbi.nlm.nih.gov/ (7 February 2023)
QM9	https://paperswithcode.com/dataset/qm9 (7 February 2023)
reactome	https://reactome.org/ (7 December 2022)
repoDB	https://repodb.net/ (7 February 2023)
scRNASeqDB	https://bioinfo.uth.edu/scrnaseqdb/ (7 February 2023)
SEER	https://seer.cancer.gov/ (27 October 2022)
SIDER	http://sideeffects.embl.de (7 February 2023)
STITCH	http://stitch.embl.de/ (7 February 2023)
STRING	https://string-db.org/ (7 February 2023)
SuperTarget	http://insilico.charite.de/supertarget/ (7 February 2023)
SynLethDB	http://synlethdb.sist.shanghaitech.edu.cn/v2/#/ (14 October 2022)
TCGA	https://portal.gdc.cancer.gov/ (10 January 2023)
TCRD	http://juniper.health.unm.edu/tcrd (7 February 2023)
TTD	https://db.idrblab.net/ttd/ (29 September 2021)
UniProt	https://www.uniprot.org/ (7 February 2023)
ZINC	http://zinc15.docking.org/ (7 February 2023)

**Table 8:** Some of the AI-designed anti-cancer drugs that have successfully entered human phase 2/3 clinical trials in the last 5 years.

Name	Chemical Structure	Company	Therapeutic Area	Target/Function	Phase
REC-2282	** 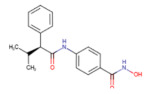 **	Recursion	Neurofibromatosis Type 2	HDAC	Phase 2/3
RLY-4008	not disclosed	Relay Therapeutics	Solid tumor	FGFR	Phase 2
BPM31510	** 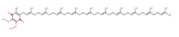 **	Berg	Solid tumor	Protein cbcl2 modulators	Phase 2
EXS-21546	not disclosed	Exscientia	Solid tumor	A2aR	Phase 1
PHI-101	** 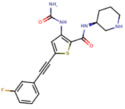 **	Pharos iBio	Ovarian cancer Breast cancer	Flt3 tyrosine kinase inhibitor	Phase 1

## Data Availability

No new data were created or analyzed in this study. Data sharing is not applicable to this article.
